# Cyclo­benzaprinium salicylate

**DOI:** 10.1107/S1600536811020642

**Published:** 2011-06-04

**Authors:** Hoong-Kun Fun, Chin Sing Yeap, M. S. Siddegowda, H. S. Yathirajan, B. Narayana

**Affiliations:** a X-ray Crystallography Unit, School of Physics, Universiti Sains Malaysia, 11800 USM, Penang, Malaysia; b Department of Studies in Chemistry, University of Mysore, Manasagangotri, Mysore 570 006, India; c Department of Studies in Chemistry, Mangalore University, Mangalagangotri 574 199, India

## Abstract

In the title mol­ecular salt [systematic name: 3-(5*H*-di­benzo[*a*,*d*]cyclo­hepten-5-yl­idene)-*N*,*N*-dimethyl-1-propanaminium 2-hy­droxy­benzoate], C_20_H_22_N^+^·C_7_H_5_O_3_^−^, the benzene rings of the cyclo­benzaprinium cation are inclined with a dihedral angle of 61.66 (7)°. An intra­molecular O—H⋯O hydrogen bond occurs within the salicylate anion, generating an *S*(6) ring. In the crystal, the cation and anion are linked by an N—H⋯O inter­action.

## Related literature

For background to cyclo­benzaprine, see: Commissiong *et al.* (1981 ▶); Katz & Dube (1988 ▶); Cimolai (2009 ▶). For related structures, see: Bindya *et al.*, (2007 ▶); Hemamalini & Fun (2010 ▶); Kolev *et al.* (2009 ▶); Thanigaimani *et al.* (2007 ▶). For graph-set notation, see: Bernstein *et al.* (1995 ▶).
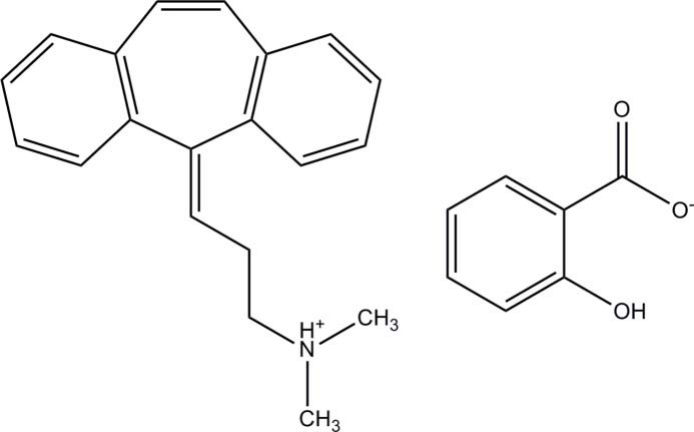


## Experimental

### 

#### Crystal data

C_20_H_22_N^+^·C_7_H_5_O_3_^−^
*M* *_r_* = 413.50
Triclinic, 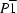

*a* = 7.4700 (8) Å
*b* = 10.8408 (12) Å
*c* = 14.9724 (16) Å
α = 76.073 (2)°
β = 77.357 (1)°
γ = 72.574 (2)°
*V* = 1108.6 (2) Å^3^
*Z* = 2
Mo *K*α radiation
μ = 0.08 mm^−1^
*T* = 296 K
0.37 × 0.21 × 0.15 mm

#### Data collection

Bruker APEXII DUO CCD diffractometer
Absorption correction: multi-scan (*SADABS*; Bruker, 2009 ▶) *T*_min_ = 0.971, *T*_max_ = 0.988
22974 measured reflections
6461 independent reflections
4411 reflections with *I* > 2σ(*I*)
*R*_int_ = 0.028

#### Refinement

*R*[*F* ^2^ > 2σ(*F* ^2^)] = 0.048
*wR*(*F* ^2^) = 0.134
*S* = 1.04
6461 reflections
280 parameters
H-atom parameters constrained
Δρ_max_ = 0.21 e Å^−3^
Δρ_min_ = −0.18 e Å^−3^

### 

Data collection: *APEX2* (Bruker, 2009 ▶); cell refinement: *SAINT* (Bruker, 2009 ▶); data reduction: *SAINT*; program(s) used to solve structure: *SHELXTL* (Sheldrick, 2008 ▶); program(s) used to refine structure: *SHELXTL*; molecular graphics: *SHELXTL*; software used to prepare material for publication: *SHELXTL* and *PLATON* (Spek, 2009 ▶).

## Supplementary Material

Crystal structure: contains datablock(s) global, I. DOI: 10.1107/S1600536811020642/hb5897sup1.cif

Structure factors: contains datablock(s) I. DOI: 10.1107/S1600536811020642/hb5897Isup2.hkl

Supplementary material file. DOI: 10.1107/S1600536811020642/hb5897Isup3.cml

Additional supplementary materials:  crystallographic information; 3D view; checkCIF report

## Figures and Tables

**Table 1:** Hydrogen-bond geometry (Å, °)

*D*—H⋯*A*	*D*—H	H⋯*A*	*D*⋯*A*	*D*—H⋯*A*
N1—H1N1⋯O1	0.91	1.75	2.6439 (16)	167
O3—H1O3⋯O2	0.99	1.55	2.4890 (19)	157

**Fig. 1.**
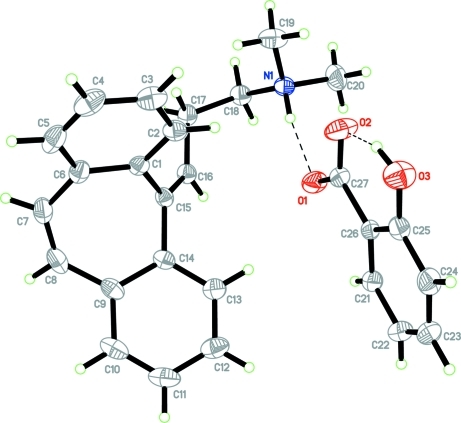
The molecular structure of the title compound, with 30% probability ellipsoids for non-H atoms. Hydrogen bonds (dashed lines) are shown.

## supplementary crystallographic information

### Comment

Cyclobenzaprine is a muscle relaxant medication used to relieve skeletal muscle spasms and associated pain in acute musculoskeletal conditions. It is the most well studied drug for this application and it also has been used off-label for fibromyalgia treatment. Cyclobenzaprine has been considered structurally related to the first-generation tricyclic antidepressants (Commissiong *et al.*, 1981; Katz & Dube, 1988; Cimolai, 2009). The crystal structures of amitriptylinium picrate (Bindya *et al.*, 2007), benzamidinium salicylate (Kolev *et al.*, 2009), 2-amino-5-chloropyridinium salicylate (Hemamalini & Fun, 2010) and 2-amino-4,6-dimethoxypyrimidinium salicylate (Thanigaimani *et al.*, 2007) have been reported.  We now report the crystal structure of the title compound, C_20_H_22_N^+^.C_7_H_5_O_3_^-^.

The asymmetric unit of the title compound consists of one cyclobenzaprinium cation and one salicylate anion (Fig. 1). The hydrogen atom of the carboxylic acid COOH group is deprotonated to the N1 atom. The two fused benzene rings of the cation make a dihedral angle of 61.66 (7)° whereas the salicylate anion is almost planar with maximum deviation of 0.063 (1) Å for atom O1. The aminium atom adopts a pyramidal conformation. The cation and anion are interconnected by N1—H1N1···O1 interaction (Table 1). An intramolecular O3—H1O3···O2 hydrogen bond (Table 1) stabilize the molecular structure of the anion molecule generating S(6) ring motif (Bernstein *et al.*, 1995).

### Experimental

Cyclobenzaprine (10 g, 0.04 mol) and salicylic acid (5.6 g, 0.04 mol) were dissolved in 50 ml of dichloromethane taken in a 100 ml round bottomed flask. Dichloromethane was distilled off under vacuum and 50 ml of ethyl acetate was added and then the flask was cooled to 278–283 K. The product formed was filtered and re-crystallized from dichloromethane to yield colourless blocks of (I) (*M*.p.: 415–416 K).

### Refinement

The O-bound and N-bound hydrogen atoms were located from difference Fourier map and refined as riding on their parent atom, with *U*_iso_(H) = 1.2 or 1.5 *U*_eq_(N or O). The rest of the hydrogen atoms were positioned geomatrically [C–H = 0.93–0.97 Å] and refined using a riding model, with *U*_iso_(H) = 1.2 or 1.5 *U*_eq_(C).

### Crystal data

**Table:**

C_20_H_22_N^+^·C_7_H_5_O_3_^−^	*Z* = 2
*M**_r_* = 413.50	*F*(000) = 440
Triclinic, *P*1	*D*_x_ = 1.239 Mg m^−^^3^
Hall symbol: -P 1	Mo *K*α radiation, λ = 0.71073 Å
*a* = 7.4700 (8) Å	Cell parameters from 5425 reflections
*b* = 10.8408 (12) Å	θ = 2.2–29.5°
*c* = 14.9724 (16) Å	µ = 0.08 mm^−^^1^
α = 76.073 (2)°	*T* = 296 K
β = 77.357 (1)°	Block, colourless
γ = 72.574 (2)°	0.37 × 0.21 × 0.15 mm
*V* = 1108.6 (2) Å^3^	

### Data collection

**Table:**

Bruker APEXII DUO CCD diffractometer	6461 independent reflections
Radiation source: fine-focus sealed tube	4411 reflections with *I* > 2σ(*I*)
graphite	*R*_int_ = 0.028
φ and ω scans	θ_max_ = 30.1°, θ_min_ = 2.0°
Absorption correction: multi-scan (*SADABS*; Bruker, 2009)	*h* = −10→10
*T*_min_ = 0.971, *T*_max_ = 0.988	*k* = −15→15
22974 measured reflections	*l* = −21→21

### Refinement

**Table:**

Refinement on *F*^2^	Primary atom site location: structure-invariant direct methods
Least-squares matrix: full	Secondary atom site location: difference Fourier map
*R*[*F*^2^ > 2σ(*F*^2^)] = 0.048	Hydrogen site location: inferred from neighbouring sites
*wR*(*F*^2^) = 0.134	H-atom parameters constrained
*S* = 1.04	*w* = 1/[σ^2^(*F*_o_^2^) + (0.0573*P*)^2^ + 0.147*P*] where *P* = (*F*_o_^2^ + 2*F*_c_^2^)/3
6461 reflections	(Δ/σ)_max_ < 0.001
280 parameters	Δρ_max_ = 0.21 e Å^−^^3^
0 restraints	Δρ_min_ = −0.18 e Å^−^^3^

### Special details

**Table:**

Geometry. All e.s.d.'s (except the e.s.d. in the dihedral angle between two l.s. planes) are estimated using the full covariance matrix. The cell e.s.d.'s are taken into account individually in the estimation of e.s.d.'s in distances, angles and torsion angles; correlations between e.s.d.'s in cell parameters are only used when they are defined by crystal symmetry. An approximate (isotropic) treatment of cell e.s.d.'s is used for estimating e.s.d.'s involving l.s. planes.
Refinement. Refinement of *F*^2^ against ALL reflections. The weighted *R*-factor *wR* and goodness of fit *S* are based on *F*^2^, conventional *R*-factors *R* are based on *F*, with *F* set to zero for negative *F*^2^. The threshold expression of *F*^2^ > σ(*F*^2^) is used only for calculating *R*-factors(gt) *etc*. and is not relevant to the choice of reflections for refinement. *R*-factors based on *F*^2^ are statistically about twice as large as those based on *F*, and *R*- factors based on ALL data will be even larger.

### Fractional atomic coordinates and isotropic or equivalent isotropic displacement parameters (Å^2^)

**Table:**

	*x*	*y*	*z*	*U*_iso_*/*U*_eq_	
N1	−0.33126 (15)	0.09099 (10)	0.18470 (7)	0.0423 (2)	
H1N1	−0.2275	0.0547	0.2133	0.051*	
C1	0.05014 (15)	0.33798 (12)	0.18708 (8)	0.0361 (3)	
C2	0.10142 (17)	0.26220 (14)	0.11781 (9)	0.0431 (3)	
H2A	0.0675	0.1833	0.1295	0.052*	
C3	0.2020 (2)	0.30282 (17)	0.03200 (10)	0.0564 (4)	
H3A	0.2353	0.2513	−0.0136	0.068*	
C4	0.2527 (2)	0.41904 (18)	0.01409 (11)	0.0640 (4)	
H4A	0.3199	0.4464	−0.0437	0.077*	
C5	0.2045 (2)	0.49480 (15)	0.08118 (12)	0.0590 (4)	
H5A	0.2374	0.5743	0.0677	0.071*	
C6	0.10616 (17)	0.45507 (12)	0.17007 (10)	0.0438 (3)	
C7	0.0743 (2)	0.53280 (14)	0.24161 (12)	0.0537 (4)	
H7A	0.0442	0.6237	0.2222	0.064*	
C8	0.0834 (2)	0.48787 (15)	0.33202 (12)	0.0557 (4)	
H8A	0.0614	0.5510	0.3684	0.067*	
C9	0.12425 (18)	0.35065 (14)	0.38045 (9)	0.0457 (3)	
C10	0.2302 (2)	0.31229 (19)	0.45369 (11)	0.0607 (4)	
H10A	0.2667	0.3758	0.4723	0.073*	
C11	0.2813 (2)	0.1843 (2)	0.49850 (11)	0.0669 (5)	
H11A	0.3535	0.1613	0.5462	0.080*	
C12	0.2254 (2)	0.08934 (17)	0.47281 (10)	0.0594 (4)	
H12A	0.2608	0.0020	0.5028	0.071*	
C13	0.11665 (18)	0.12385 (14)	0.40225 (9)	0.0457 (3)	
H13A	0.0782	0.0596	0.3857	0.055*	
C14	0.06458 (16)	0.25355 (13)	0.35607 (8)	0.0376 (3)	
C15	−0.05187 (16)	0.28950 (11)	0.28004 (8)	0.0347 (2)	
C16	−0.22561 (17)	0.27224 (12)	0.29535 (9)	0.0392 (3)	
H16A	−0.2678	0.2322	0.3553	0.047*	
C17	−0.36111 (17)	0.30986 (13)	0.22713 (10)	0.0436 (3)	
H17A	−0.2928	0.3319	0.1650	0.052*	
H17B	−0.4586	0.3884	0.2404	0.052*	
C18	−0.45677 (17)	0.20412 (13)	0.22789 (10)	0.0434 (3)	
H18A	−0.5051	0.1707	0.2919	0.052*	
H18B	−0.5648	0.2441	0.1951	0.052*	
C19	−0.2757 (2)	0.12856 (18)	0.08299 (10)	0.0621 (4)	
H19A	−0.1936	0.0531	0.0588	0.093*	
H19B	−0.2101	0.1962	0.0704	0.093*	
H19C	−0.3874	0.1608	0.0537	0.093*	
C20	−0.4273 (3)	−0.01685 (17)	0.20561 (14)	0.0736 (5)	
H20A	−0.3458	−0.0895	0.1780	0.110*	
H20B	−0.5440	0.0143	0.1805	0.110*	
H20C	−0.4540	−0.0450	0.2719	0.110*	
O1	−0.05304 (14)	−0.04235 (10)	0.28085 (7)	0.0564 (3)	
O2	0.10069 (17)	−0.09085 (12)	0.14508 (7)	0.0724 (4)	
O3	0.42092 (17)	−0.24876 (12)	0.13184 (7)	0.0674 (3)	
H1O3	0.2980	−0.1850	0.1204	0.101*	
C21	0.22965 (19)	−0.23076 (12)	0.37511 (9)	0.0433 (3)	
H21A	0.1221	−0.1879	0.4115	0.052*	
C22	0.3758 (2)	−0.32023 (15)	0.41757 (11)	0.0570 (4)	
H22A	0.3665	−0.3378	0.4821	0.068*	
C23	0.5356 (2)	−0.38335 (15)	0.36347 (12)	0.0601 (4)	
H23A	0.6346	−0.4429	0.3919	0.072*	
C24	0.5504 (2)	−0.35955 (14)	0.26882 (11)	0.0540 (4)	
H24A	0.6591	−0.4028	0.2332	0.065*	
C25	0.40337 (18)	−0.27075 (13)	0.22540 (9)	0.0429 (3)	
C26	0.24146 (16)	−0.20414 (11)	0.27883 (8)	0.0356 (2)	
C27	0.08451 (18)	−0.10603 (12)	0.23224 (9)	0.0420 (3)	

### Atomic displacement parameters (Å^2^)

**Table:**

	*U*^11^	*U*^22^	*U*^33^	*U*^12^	*U*^13^	*U*^23^
N1	0.0371 (5)	0.0432 (6)	0.0491 (6)	−0.0090 (4)	−0.0163 (4)	−0.0070 (5)
C1	0.0256 (5)	0.0387 (6)	0.0429 (6)	−0.0071 (4)	−0.0102 (4)	−0.0030 (5)
C2	0.0332 (6)	0.0510 (7)	0.0451 (7)	−0.0094 (5)	−0.0098 (5)	−0.0078 (6)
C3	0.0451 (8)	0.0727 (10)	0.0442 (7)	−0.0068 (7)	−0.0065 (6)	−0.0088 (7)
C4	0.0516 (9)	0.0745 (11)	0.0502 (9)	−0.0140 (8)	−0.0029 (7)	0.0098 (8)
C5	0.0463 (8)	0.0496 (8)	0.0722 (10)	−0.0172 (7)	−0.0128 (7)	0.0142 (7)
C6	0.0316 (6)	0.0389 (6)	0.0579 (8)	−0.0086 (5)	−0.0121 (5)	0.0002 (5)
C7	0.0446 (7)	0.0371 (7)	0.0827 (11)	−0.0119 (6)	−0.0166 (7)	−0.0098 (7)
C8	0.0485 (8)	0.0519 (8)	0.0783 (11)	−0.0163 (6)	−0.0111 (7)	−0.0290 (7)
C9	0.0358 (6)	0.0596 (8)	0.0490 (7)	−0.0170 (6)	−0.0041 (5)	−0.0208 (6)
C10	0.0550 (9)	0.0886 (12)	0.0540 (9)	−0.0301 (8)	−0.0129 (7)	−0.0239 (8)
C11	0.0573 (9)	0.1065 (14)	0.0440 (8)	−0.0310 (9)	−0.0176 (7)	−0.0063 (8)
C12	0.0522 (8)	0.0754 (10)	0.0450 (8)	−0.0196 (8)	−0.0119 (6)	0.0066 (7)
C13	0.0402 (7)	0.0547 (8)	0.0419 (7)	−0.0172 (6)	−0.0048 (5)	−0.0039 (6)
C14	0.0284 (5)	0.0493 (7)	0.0371 (6)	−0.0126 (5)	−0.0017 (4)	−0.0119 (5)
C15	0.0297 (5)	0.0345 (6)	0.0422 (6)	−0.0094 (4)	−0.0059 (4)	−0.0101 (5)
C16	0.0330 (6)	0.0407 (6)	0.0452 (7)	−0.0120 (5)	−0.0054 (5)	−0.0081 (5)
C17	0.0306 (6)	0.0403 (6)	0.0607 (8)	−0.0071 (5)	−0.0134 (5)	−0.0080 (6)
C18	0.0264 (5)	0.0502 (7)	0.0557 (7)	−0.0107 (5)	−0.0092 (5)	−0.0105 (6)
C19	0.0550 (9)	0.0819 (11)	0.0481 (8)	−0.0085 (8)	−0.0176 (7)	−0.0131 (7)
C20	0.0793 (12)	0.0570 (10)	0.1005 (14)	−0.0321 (9)	−0.0296 (10)	−0.0127 (9)
O1	0.0484 (6)	0.0553 (6)	0.0579 (6)	0.0085 (5)	−0.0183 (5)	−0.0156 (5)
O2	0.0730 (8)	0.0825 (8)	0.0445 (6)	0.0097 (6)	−0.0213 (5)	−0.0066 (5)
O3	0.0636 (7)	0.0813 (8)	0.0473 (6)	−0.0044 (6)	0.0015 (5)	−0.0210 (5)
C21	0.0460 (7)	0.0404 (7)	0.0433 (7)	−0.0075 (5)	−0.0102 (5)	−0.0093 (5)
C22	0.0682 (10)	0.0537 (8)	0.0500 (8)	−0.0087 (7)	−0.0267 (7)	−0.0049 (6)
C23	0.0536 (9)	0.0471 (8)	0.0803 (11)	0.0029 (7)	−0.0348 (8)	−0.0120 (7)
C24	0.0399 (7)	0.0470 (8)	0.0751 (10)	−0.0003 (6)	−0.0117 (7)	−0.0229 (7)
C25	0.0418 (7)	0.0405 (7)	0.0488 (7)	−0.0107 (5)	−0.0066 (5)	−0.0133 (5)
C26	0.0357 (6)	0.0306 (5)	0.0424 (6)	−0.0084 (4)	−0.0108 (5)	−0.0069 (5)
C27	0.0438 (7)	0.0370 (6)	0.0460 (7)	−0.0069 (5)	−0.0159 (5)	−0.0059 (5)

### Geometric parameters (Å, °)

**Table:**

N1—C19	1.4775 (18)	C14—C15	1.4904 (16)
N1—C20	1.4855 (19)	C15—C16	1.3291 (16)
N1—C18	1.4930 (17)	C16—C17	1.4963 (17)
N1—H1N1	0.9057	C16—H16A	0.9300
C1—C2	1.3947 (18)	C17—C18	1.5184 (18)
C1—C6	1.4026 (17)	C17—H17A	0.9700
C1—C15	1.4892 (17)	C17—H17B	0.9700
C2—C3	1.3828 (19)	C18—H18A	0.9700
C2—H2A	0.9300	C18—H18B	0.9700
C3—C4	1.372 (2)	C19—H19A	0.9600
C3—H3A	0.9300	C19—H19B	0.9600
C4—C5	1.369 (2)	C19—H19C	0.9600
C4—H4A	0.9300	C20—H20A	0.9600
C5—C6	1.408 (2)	C20—H20B	0.9600
C5—H5A	0.9300	C20—H20C	0.9600
C6—C7	1.455 (2)	O1—C27	1.2457 (16)
C7—C8	1.333 (2)	O2—C27	1.2586 (16)
C7—H7A	0.9300	O3—C25	1.3468 (16)
C8—C9	1.462 (2)	O3—H1O3	0.9910
C8—H8A	0.9300	C21—C22	1.3819 (18)
C9—C10	1.4017 (19)	C21—C26	1.3888 (17)
C9—C14	1.4061 (18)	C21—H21A	0.9300
C10—C11	1.367 (3)	C22—C23	1.380 (2)
C10—H10A	0.9300	C22—H22A	0.9300
C11—C12	1.378 (2)	C23—C24	1.364 (2)
C11—H11A	0.9300	C23—H23A	0.9300
C12—C13	1.3855 (19)	C24—C25	1.3907 (19)
C12—H12A	0.9300	C24—H24A	0.9300
C13—C14	1.3887 (19)	C25—C26	1.3984 (17)
C13—H13A	0.9300	C26—C27	1.4988 (16)
			
C19—N1—C20	110.16 (12)	C1—C15—C14	113.33 (9)
C19—N1—C18	112.70 (11)	C15—C16—C17	127.45 (12)
C20—N1—C18	109.75 (12)	C15—C16—H16A	116.3
C19—N1—H1N1	110.9	C17—C16—H16A	116.3
C20—N1—H1N1	103.5	C16—C17—C18	114.56 (11)
C18—N1—H1N1	109.5	C16—C17—H17A	108.6
C2—C1—C6	119.35 (12)	C18—C17—H17A	108.6
C2—C1—C15	119.75 (11)	C16—C17—H17B	108.6
C6—C1—C15	120.77 (11)	C18—C17—H17B	108.6
C3—C2—C1	120.88 (14)	H17A—C17—H17B	107.6
C3—C2—H2A	119.6	N1—C18—C17	114.71 (10)
C1—C2—H2A	119.6	N1—C18—H18A	108.6
C4—C3—C2	120.01 (15)	C17—C18—H18A	108.6
C4—C3—H3A	120.0	N1—C18—H18B	108.6
C2—C3—H3A	120.0	C17—C18—H18B	108.6
C5—C4—C3	120.11 (14)	H18A—C18—H18B	107.6
C5—C4—H4A	119.9	N1—C19—H19A	109.5
C3—C4—H4A	119.9	N1—C19—H19B	109.5
C4—C5—C6	121.47 (15)	H19A—C19—H19B	109.5
C4—C5—H5A	119.3	N1—C19—H19C	109.5
C6—C5—H5A	119.3	H19A—C19—H19C	109.5
C1—C6—C5	118.11 (13)	H19B—C19—H19C	109.5
C1—C6—C7	122.59 (12)	N1—C20—H20A	109.5
C5—C6—C7	119.24 (13)	N1—C20—H20B	109.5
C8—C7—C6	127.09 (13)	H20A—C20—H20B	109.5
C8—C7—H7A	116.5	N1—C20—H20C	109.5
C6—C7—H7A	116.5	H20A—C20—H20C	109.5
C7—C8—C9	127.38 (13)	H20B—C20—H20C	109.5
C7—C8—H8A	116.3	C25—O3—H1O3	102.1
C9—C8—H8A	116.3	C22—C21—C26	120.81 (13)
C10—C9—C14	117.85 (14)	C22—C21—H21A	119.6
C10—C9—C8	119.14 (13)	C26—C21—H21A	119.6
C14—C9—C8	123.00 (12)	C23—C22—C21	119.49 (14)
C11—C10—C9	121.92 (15)	C23—C22—H22A	120.3
C11—C10—H10A	119.0	C21—C22—H22A	120.3
C9—C10—H10A	119.0	C24—C23—C22	120.90 (13)
C10—C11—C12	119.78 (14)	C24—C23—H23A	119.5
C10—C11—H11A	120.1	C22—C23—H23A	119.5
C12—C11—H11A	120.1	C23—C24—C25	120.11 (13)
C11—C12—C13	120.04 (15)	C23—C24—H24A	119.9
C11—C12—H12A	120.0	C25—C24—H24A	119.9
C13—C12—H12A	120.0	O3—C25—C24	119.13 (12)
C12—C13—C14	120.62 (14)	O3—C25—C26	120.96 (12)
C12—C13—H13A	119.7	C24—C25—C26	119.91 (12)
C14—C13—H13A	119.7	C21—C26—C25	118.77 (11)
C13—C14—C9	119.76 (12)	C21—C26—C27	121.09 (11)
C13—C14—C15	120.17 (11)	C25—C26—C27	120.13 (11)
C9—C14—C15	120.06 (11)	O1—C27—O2	123.33 (12)
C16—C15—C1	124.75 (11)	O1—C27—C26	118.89 (11)
C16—C15—C14	121.83 (11)	O2—C27—C26	117.77 (12)
			
C6—C1—C2—C3	−1.77 (17)	C6—C1—C15—C16	118.97 (14)
C15—C1—C2—C3	−177.74 (11)	C2—C1—C15—C14	111.50 (12)
C1—C2—C3—C4	0.0 (2)	C6—C1—C15—C14	−64.41 (14)
C2—C3—C4—C5	0.2 (2)	C13—C14—C15—C16	61.58 (16)
C3—C4—C5—C6	1.3 (2)	C9—C14—C15—C16	−119.26 (14)
C2—C1—C6—C5	3.19 (17)	C13—C14—C15—C1	−115.15 (12)
C15—C1—C6—C5	179.12 (11)	C9—C14—C15—C1	64.01 (14)
C2—C1—C6—C7	−173.92 (11)	C1—C15—C16—C17	−7.0 (2)
C15—C1—C6—C7	2.01 (17)	C14—C15—C16—C17	176.69 (11)
C4—C5—C6—C1	−3.0 (2)	C15—C16—C17—C18	134.27 (14)
C4—C5—C6—C7	174.19 (13)	C19—N1—C18—C17	−67.76 (14)
C1—C6—C7—C8	36.3 (2)	C20—N1—C18—C17	169.07 (12)
C5—C6—C7—C8	−140.80 (16)	C16—C17—C18—N1	−73.67 (15)
C6—C7—C8—C9	−1.2 (2)	C26—C21—C22—C23	0.3 (2)
C7—C8—C9—C10	144.17 (16)	C21—C22—C23—C24	−0.6 (2)
C7—C8—C9—C14	−34.8 (2)	C22—C23—C24—C25	0.0 (2)
C14—C9—C10—C11	2.4 (2)	C23—C24—C25—O3	−179.97 (14)
C8—C9—C10—C11	−176.58 (15)	C23—C24—C25—C26	1.1 (2)
C9—C10—C11—C12	−1.1 (3)	C22—C21—C26—C25	0.73 (19)
C10—C11—C12—C13	−0.5 (2)	C22—C21—C26—C27	−179.26 (13)
C11—C12—C13—C14	0.7 (2)	O3—C25—C26—C21	179.66 (12)
C12—C13—C14—C9	0.62 (19)	C24—C25—C26—C21	−1.40 (19)
C12—C13—C14—C15	179.78 (12)	O3—C25—C26—C27	−0.35 (19)
C10—C9—C14—C13	−2.13 (18)	C24—C25—C26—C27	178.59 (12)
C8—C9—C14—C13	176.83 (12)	C21—C26—C27—O1	4.18 (19)
C10—C9—C14—C15	178.71 (12)	C25—C26—C27—O1	−175.80 (12)
C8—C9—C14—C15	−2.33 (19)	C21—C26—C27—O2	−176.97 (13)
C2—C1—C15—C16	−65.12 (16)	C25—C26—C27—O2	3.04 (19)

### Hydrogen-bond geometry (Å, °)

**Table:**

*D*—H···*A*	*D*—H	H···*A*	*D*···*A*	*D*—H···*A*
N1—H1N1···O1	0.91	1.75	2.6439 (16)	167
O3—H1O3···O2	0.99	1.55	2.4890 (19)	157
